# EcoStack-Pro: an adaptive federated learning framework for interpretable ESG auditing across heterogeneous industrial sectors

**DOI:** 10.3389/frai.2026.1813511

**Published:** 2026-05-13

**Authors:** Md. Abul Kalam Azad, Abdul Kadar Muhammad Masum, Md. Abdur Rahman, Md. Tofael Ahmed Bhuiyan, Farzan Majeed Noori, Md Zia Uddin

**Affiliations:** 1 Department of Business and Technology Management, Islamic University of Technology, Gazipur, Bangladesh; 2 Department of Computer Science and Engineering, Southeast University, Dhaka, Bangladesh; 3 Computational Intelligence Lab, Southeast University, Dhaka, Bangladesh; 4 Department of Informatics, University of Oslo, Oslo, Norway; 5 Sustainable Communication Technologies Department, SINTEF Digital, Oslo, Norway

**Keywords:** adaptive aggregation, corporate sustainability, EcoStack-Pro, ESG auditing, explainable AI (XAI), federated learning, gradient boosting ensembles

## Abstract

**Introduction:**

The paradigm shifts toward environmental, social, and governance (ESG) metrics has necessitated advanced auditing systems capable of analyzing complex, non-financial performance indicators. However, traditional centralized artificial intelligence (AI) models conflict with increasingly stringent data privacy regulations, while conventional federated learning approaches struggle to converge under the high statistical heterogeneity and data imbalance typical of diverse industrial sectors.

**Methods:**

To address the trade-off between high-precision forecasting and data sovereignty, this study proposes EcoStack-Pro, a decentralized auditing framework driven by a stacked ensemble of LightGBM, XGBoost, and Gradient Boosting regressors, optimized via a Bayesian ridge meta-learner. Central to this architecture is the Fed-GenAdaptive algorithm, which employs a soft-gating mechanism with softmax normalization to dynamically weight client contributions according to their local validation errors and generalization gaps.

**Results:**

Utilizing a stratified dataset of 21,400 firm-year observations across 10 distinct industrial clients, the framework achieves a test-set R2 of 0.9614. This performance retains 98.2% of the predictive power of the centralized upper bound (R2 of 0.9790) while strictly preserving corporate privacy.

**Discussion:**

Furthermore, the integration of Shapley additive explanations (SHAP) and local interpretable model-agnostic explanations (LIME) enhances model interpretability, elucidating the non-linear drivers of governance ratings. These results demonstrate that adaptive, diverse ensemble strategies can overcome the limitations of single-model federated baselines, providing a robust framework for secure, cross-sector sustainable finance auditing.

## 1 Introduction

Corporate sustainability has increasingly shifted toward stringent ESG auditing frameworks. Consequently, modern environmental standards are now closely aligned with the core commitments of the Paris Agreement (Kim, 2024). Social dimensions encompass human rights and the accountability of stakeholders (Simmons et al., 2024). Traditional audit approaches find it difficult to handle large volumes of unstructured data. Artificial intelligence (AI) and sophisticated analytics are needed in current practice. Extensive data extraction from various sources is now performed with the assistance of AI systems (Ullah et al., 2024). The use of real-time feedback systems helps address limitations in traditional batch-processing design. Industry 4.0 technologies use advanced algorithms to provide opportunities for strategic decision-making. Machine learning is also necessary to effectively identify and mitigate risks. These advanced systems ensure that corporations are resilient and not solely dependent on short-term financial performance indicators (Dasinapa and Ermawati, 2024).

Advanced machine learning algorithms are currently able to isolate alpha associated with ESG factors (Carlei et al., 2025). Deep learning models are more effective than traditional approaches in making predictions (Zhang et al., 2025). Convolutional neural networks and long short-term memory networks are used to improve predictions. ESG ratings have become more proactive rather than reactive. Automated systems anticipate potential compliance threats even before they materialize. Nonetheless, data privacy laws present significant bottlenecks to model development. Strict data protection laws do not allow the centralization of proprietary information worldwide. Financial fraud can be detected by neural networks, but privacy limitations remain (Ilori et al., 2024). Cross-sector ESG auditing is significantly impaired by statistical heterogeneity. Universal benchmarking and data sovereignty are now supported by federated learning architectures. Decentralized models allow robust global model training in the absence of centralization. Privacy-preserving technologies enhance ESG audit systems.

Corporate sustainability incorporates ESG aspects in a unified manner (Gu et al., 2023). The environmental factor centers on carbon footprints and emission reduction. Green practices support the standards of the Paris Agreement developed at the international level (Kim, 2024). Social dimensions broadly focus on human rights in the corporate supply chain. Corporate responsibility toward stakeholders demonstrates accountability to society (Simmons et al., 2024). Another important aspect of the social dimension is community integration. Governance aspects ensure institutional transparency mechanisms and board diversity. Data security governance significantly improves decision-making processes at all levels within the organization (Rakipi and D'Onza, 2024). ESG measures evaluate the potential of sustainable development that is not measured in financial terms. The paradigm shift emphasizes long-term corporate resilience rather than short-term benefits. Climate change and global social equity challenges escalate the reporting imperative. These complex non-financial performance indicators should be systematically integrated into modern audits.

ESG audits represent a significant paradigm shift compared with traditional financial auditing (Boiral et al., 2024). Assessment is conducted on non-financial performance measures based on diverse data sources. Critical audit evidence includes satellite imagery and social media analysis. Conventional approaches have a significant problem combining multi-source unstructured data. Environmental risk pathways and feedback cannot be modeled using fixed structures (Sheehan et al., 2024). Traditional AI models do not have sequential adaptive decision-making abilities. Convolutional Neural Networks process environmental datasets holistically (Rüdele and Wolf, 2024). SSM networks process social data streams effectively. These models are unable to dynamically adapt to audit mechanisms. Reinforcement learning has addressed these technological gaps by simulating dynamic environments. The adaptive extraction of features using algorithms derived from Q-learning is enabled by significant computational power (Ullah et al., 2024). Deep policy networks are effective in modeling complex risk transmission pathways. This enables proactive rather than reactive ESG ratings (Lenz and Hoos, 2023). Real-time rewards dynamically optimize audit resources (Asif et al., 2023). Reinforcement learning is more effective than conventional approaches.

In this study, a decentralized auditing framework called EcoStack-Pro is proposed. The proposed architecture combines gradient boosting ensembles and a Bayesian meta-learner. Fed-GenAdaptive is a new aggregation algorithm that handles high sectoral heterogeneity. Dynamic weighting schemes prioritize trusted clients through a soft-gating mechanism. Decentralized training processes ensure data privacy across industrial sectors worldwide. Experimental results show that the predictive performance is close to the centralized upper bound. Explainability modules are also integrated to provide clear insights into the complex drivers of governance performance. Robust scaling addresses outliers and missing values in financial reports. This approach captures non-linear relationships across multi-sector corporate datasets. The convergence of models using stacking techniques is also stable across imbalanced data distributions.

To summarize, the main contributions of this study are as follows:

i. EcoStack-Pro architecture combines three specialized variants of gradient boosting models.
ii. The Fed-GenAdaptive algorithm optimizes global aggregation weights through validation error metrics.
iii. Softmax normalization converts penalty scores into adaptive and robust global weights.
iv. Bayesian ridge meta-learner guarantees sound convergence on multi-sector data.
v. Federated systems eliminate data silos while maintaining strict corporate privacy standards.
vi. Shapley additive explanations (SHAP) and local interpretable model-agnostic explanations (LIME) tools deliver instance-based local and global interpretations.
vii. The proposed model exceeds single-model baseline performance across 10 different industrial clients.

This study is organized into nine sections. The first two sections introduce sustainability and review the available literature. The third section describes federated learning solutions and the principal framework. The fourth section covers data engineering processes and sector partitioning. The fifth section defines the EcoStack-Pro scheme and base models. The sixth section describes the Fed-GenAdaptive global aggregation algorithm framework. The seventh section presents experimental results and comparative performance analysis. Section 8 examines model transparency using explainable artificial intelligence (XAI) techniques. The ninth section provides concluding remarks and future work. This systematic arrangement provides a logical sequence of investigation.

## 2 Literature review

The initial ESG auditing studies investigated strategic influence and new audit technology (Dasinapa and Ermawati, 2024). Research showed that ESG factors were of vital importance to corporations. The major discoveries have significantly enhanced the long-term competitive advantage. Organizational change driven by innovation and strategic risk mitigation processes became important. Another important success factor was strategic stakeholder engagement. ESG should be integrated into the standard organizational decision-making system. International analytical models were used to examine regulatory variations in a holistic manner (Singhania and Saini, 2023). It was found that there was a significant difference in ESG disclosure among corporations. Substantial differences were observed between countries with varying regulatory regimes. Governance structures and competitive market forces had a major influence on reporting practices. Standardized auditing rules would enhance the efficiency and transparency of information globally. Coherent reporting of data addresses cross-border comparability issues. ESG assurance models need to be standardized across countries.

The quality of disclosure and global standardization are receiving increased attention in ESG studies. Substantial assessment of reporting standards and organizational integrity has been conducted through extensive data analysis (Del Giudice and Rigamonti, 2020). Disclosure practices are largely dependent on organizational scale as well as industry-specific factors. The mechanisms driving voluntary ESG disclosure include external stakeholder pressures. The credibility of third-party assurance was determined using comprehensive research findings (Lenz and Chesshire, 2023). External audits substantially enhance the credibility of reported ESG data. There is a strong need for assurance functions, both as a theoretical and practical requirement. Core ESG systems should be embedded with assurance mechanisms. These provide relevant evidence for verifying complex non-financial information. Assurance addresses growing concerns about potential large-scale greenwashing practices. It establishes stakeholder confidence in corporate sustainability claims. Regulatory oversight represents a significant advancement in ESG ecosystems. Autonomous assurance frameworks may be required in future ESG structures.

The traditional auditing theory provides the ground rules that can be used in ESG auditing. Critical audit risk frameworks also deal with regulatory settings dynamics (Susanto and Kalsum, 2023). Inherent risk, control risk, and detection risk are included in risk assessment. This empirical paradigm provides ESG audit practices with a high degree of rigor. The research conducted on audit sampling optimization identified strategies to enhance auditing efficiency (Minkkinen et al., 2024). Quality assurance was maintained at the forefront, despite significant professional resource limitations. These methodological contributions can provide significant guidance to ESG practitioners. They can manage both audit coverage and available useful resources effectively. The development of theory helps in establishing a fair balance between methodological rigor and practical feasibility. ESG auditing frameworks successfully incorporate traditional auditing strengths. With this combination, it is possible to assess complex non-financial data effectively. Finally, tested methodologies enable sustainability assurance procedures holistically.

Information technology developments have contributed to the advancement of data mining in auditing. This method extrapolates latent audit evidence from financial data (Zou, 2023). Anomaly detection operates within multifaceted, multi-source data ecosystems that are typical of ESG. Data-driven audit risk assessment models contribute to an increased level of objectivity (Pangastuti, 2023). Quantitative analysis offers a scientific basis for more rigorous auditing. Integrated approaches allow rigorous non-financial data querying and review. Unstructured data methods address environmental and social information issues. Actionable audit evidence is derived from social sentiment analysis and satellite imagery. These strategies address the problems of volume and diversity. Technology enhances analytical processes and makes them more reliable and comprehensive. Ensuring sustainability requires technology-based, non-manual sampling analysis. New methodologies are used to revolutionize auditing significantly.

The recent auditing studies have developed AI as a critical focus. Complex neural networks were able to improve the accuracy of analytics and fraud detection (Ilori et al., 2024). The intelligent agent technology simplified complex internal auditing processes significantly (da Silva and Imoniana, 2021). There was a high level of audit timeliness due to real-time monitoring and automated alerts. Nonetheless, AI audit research is focused largely on traditional financial areas. The current level of academic interest in green auditing is quite low. The specifics of ESG data entail analytical and verification challenges. Non-financial information cannot be analyzed using the same types of analytical methods as financial models. There are critical gaps in the application of AI to sustainability. AI tools can still be utilized to further research in sustainability assurance. This gap needs to be addressed in next-generation audit quality.

The auditing landscape is currently undergoing a paradigm shift toward the integration of privacy-preserving and XAI. Recent scholarship demonstrates that AI fundamentally improves the reliability and transparency of contemporary financial assurance (Al-Omush et al., 2025). Furthermore, decentralized collaboration across intricate, cross-enterprise supply chains is increasingly enabled by federated learning paradigms (Ravikumar and Aarthi, 2026), while the fusion of AI and blockchain technology provides robust security and trust for modern smart finance systems (Ali et al., 2026). In the specific context of sustainability, cutting-edge data mining techniques are advancing the evaluation of sustainable investments and ESG analytics (Singh, 2025). Despite these significant advancements, a critical limitation remains: existing analytical frameworks predominantly rely on homogeneous ensemble structures or opaque neural network architectures. These conventional models inherently falter when confronted with extreme non-IID data distributions characteristic of global ESG repositories. Consequently, the development of adaptive, heterogeneous ensemble weighting techniques is imperative to achieve stable and interpretable convergence in federated environments.

Conventional federated learning frameworks, including FedAvg and FedProx, are effective for addressing basic data privacy but typically rely on client data volume or raw training loss for model aggregation. When applied to multi-sector ESG datasets, which inherently exhibit extreme statistical heterogeneity and severe class imbalances, these standard approaches frequently face convergence failure and severe performance degradation. Furthermore, current federated ensemble methods generally utilize homogeneous, single-algorithm baselines rather than sophisticated multi-tier stacking architectures.

This study explicitly bridges this critical research gap through two primary methodological novelties. First, we replace standard single-model nodes with the proposed EcoStack-Pro architecture, leveraging a localized all-tree stacking ensemble guided by a Bayesian ridge meta-learner. This design effectively mitigates the convergence instability that traditional neural networks experience when processing tabular financial data. Second, we introduce the Fed-GenAdaptive aggregation algorithm, which completely discards traditional size-proportional weighting. By implementing a dynamic dual-metric soft-gating mechanism via softmax normalization, this algorithm evaluates both local validation errors and generalization gaps. Actively penalizing the generalization gap, rather than relying solely on training loss, allows the framework to dynamically filter out noisy or overfitted updates. This provides a fundamentally adaptive approach to federated weighting, ensuring robust global convergence across isolated and highly skewed industrial data environments.

## 3 Methodology

**Figure 1:**
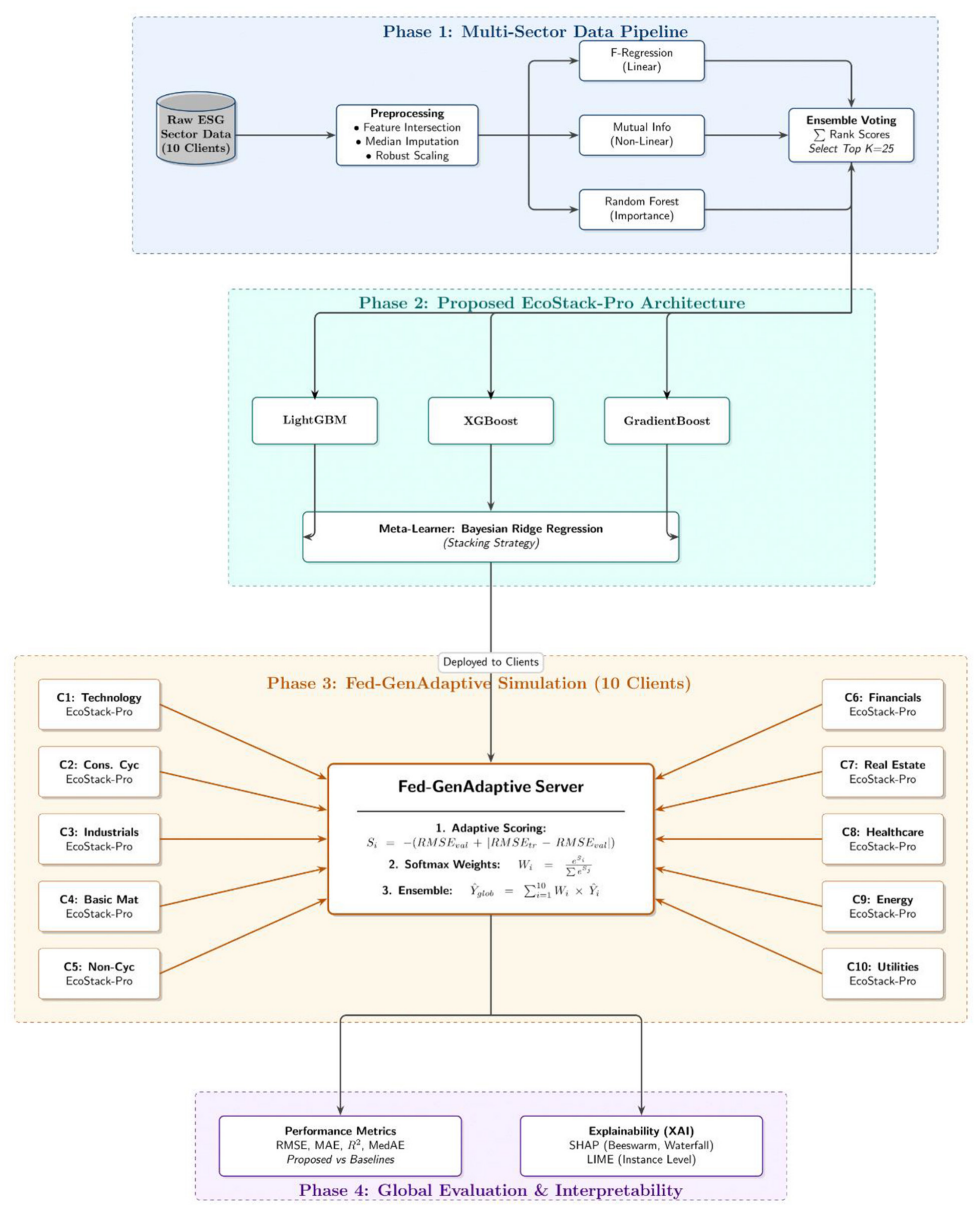
Schematic representation of the proposed EcoStack-Pro architecture and the Fed-GenAdaptive global aggregation methodology.

The federated system, grounded on a multi-sector architecture, incorporates effective local architectural designs. This dataset consists of 21,400 firm-year observations. There are 10 different industrial sectors, each split into separate federated learning clients. Each client is subjected to a stringent preprocessing pipeline to ensure consistency in feature alignment. Scaling using the interquartile range and ensemble-based feature selection determine 25 predictive features. The local EcoStack-Pro architecture makes use of specialized tree-based gradient boosting ensembles. The base learning models are LightGBM, XGBoost, and Gradient Boosting. A Bayesian ridge meta-learner is used to integrate base predictions to attain sound probabilistic convergence. The new Fed-GenAdaptive algorithm is used to aggregate models globally, as opposed to averaging. This mechanism is used to evaluate the validation error and generalization gap of each client. These scores are converted into adaptive global aggregation weights through softmax normalization. SHAP determines the key drivers of governance, and LIME estimates the boundaries of local decisions. SHAP and LIME techniques offer global and local interpretability to practitioners. The unified architecture attains near-centralized performance without compromising data privacy. Figure 1 shows the proposed methodology.

### 3.1 Data engineering and sector partitioning

This study is based on a carefully selected panel dataset that spans the years 2010 to 2022 and includes 21,400 firm-year observations from 2,481 publicly traded companies in various international jurisdictions. While ESG and corporate governance indicators were gathered from corporate disclosures, specialized ESG indices, and verified sustainability archives, financial and operational variables were gathered from the Refinitiv database. The dataset ensures industry-adjusted and jurisdiction-adjusted coverage appropriate for ESG benchmarking by covering a wide range of industrial sectors, such as consumer staples, energy, materials, and finance. Extreme outliers exceeding three standard deviations were eliminated to guarantee data integrity, and median substitution was used to impute minor missing values. To replicate a realistic federated learning environment, we strictly divide the dataset by industrial sector, in contrast to previous studies that randomly shuffle observations. Each sector operates as an independent client node, preventing cross-sector information leakage and preserving sector-specific data distributions.

### 3.2 The problem of data silos and privacy

Traditional centralized learning models frequently fail to comply with modern corporate privacy regulations. Consequently, sensitive governance metrics are often confined to isolated data silos across various industrial sectors. Such fragmented repositories impede the creation of universal predictive ESG models. Sectoral heterogeneity ensures that data distributions are non-identical. The statistical characteristics of technology companies differ significantly from those of the energy industries. Federated learning addresses these challenges through decentralized local training. Only model parameter updates are transmitted to a central server. Normal averaging methods presuppose homogeneity and size-based contribution. Large, noisy datasets may severely degrade global model quality. The quality of the model should be emphasized in effective aggregation rather than data quantity.

### 3.3 The federated learning solution

Federated learning provides a framework for preserving sensitive information. Local training is performed instead of centralized aggregation to guarantee data security. Traditional averaging techniques face challenges under high statistical heterogeneity across sectors. The proposed Fed-GenAdaptive algorithm presents intelligent and adaptive aggregation strategies. Composite client scores are based on validation errors and generalization gaps. Softmax normalization is used to convert these metrics into optimized adaptive aggregation weights. The framework prioritizes reliable clients and filters unstable contributors. Iterative training processes help stabilize global model convergence. Decentralized training processes effectively manage complex and heterogeneous industrial distributions. This system balances high predictive performance with strict data governance.

### 3.4 Contributions

This study also has three fundamental methodological contributions to federated learning. The EcoStack-Pro architecture employs three different variants of gradient boosting at the local level. A Bayesian ridge meta-learner ensures sound convergence on financial data. The Fed-GenAdaptive algorithm allocates client contributions according to model quality. Softmax transformations convert validation metrics into adaptive global weights. This reduces noise from unstable models in the network. SHAP and LIME are integrated as explainable AI methods. SHAP determines global drivers, whereas LIME provides instance-based local explanations. The framework achieves high performance while maintaining strict data privacy. The proposed methodology addresses non-linear and complex dependencies in financial data.

## 4 Data engineering and sector partitioning

This section is based on a curated multi-sector dataset spanning 13 years. Federated learning has 10 industrial clients as independent clients. Such distributions represent realistic and imbalanced corporate data silos in the real world. The strict preprocessing pipeline guarantees high-quality feature alignment among clients. This is performed to eliminate potential target leakage variables and preserve model integrity. Numeric values are filled in using the local column median to impute missing values. Robust scaling uses the interquartile range to deal with financial outliers. Predictive features are identified using statistical techniques to select 25 computationally efficient features. The robustness of the selection process is observed across different statistical and modeling assumptions. The privacy of corporate data is strictly maintained during training through data engineering processes.

### 4.1 Dataset statistics

The curated dataset has high levels of sectoral imbalance and statistical heterogeneity. More than 21,000 records belong to various corporate organizations. Federated learning includes 10 industrial clients. The volumes of industrial data reflect a significantly skewed distribution. Technology and utilities are the largest and smallest clients, respectively. The imbalance ratio is 3.5 among the participating sectors. This inherent skewness poses significant difficulties for conventional federated aggregation. Distributions are realistically determined based on data availability across industrial sectors worldwide. The structure should be able to deal with imbalance without major loss in predictive performance. Well-established engineering procedures ensure that corporate privacy is observed during training. Table 1 shows the sector-wise data distribution across federated clients.

**Table 1:** Sector-wise data distribution across federated clients.

Client ID	Sector name	Instances	Unique companies
0	Technology	3,379	377
1	Consumer cyclicals	3,306	384
2	Industrials	3,289	374
3	Basic materials	2,766	312
4	Consumer non-cyclicals	2,014	229
5	Financials	1,838	231
6	Real estate	1,788	235
7	Healthcare	1,031	119
8	Energy	1,019	131
9	Utilities	970	120
Total	Global	21,400	2,481

For all predictive models, the continuous ESG Combined Score serves as the primary target variable. Sourced from the Refinitiv database, this metric synthesizes environmental, social, and governance dimensions alongside ESG controversies to provide a holistic measure of sustainability performance. The independent feature space consists of 25 variables identified via an ensemble feature selection approach. Key operational and sustainability indicators within this set include Board Gender Diversity, CO2 Emissions, Resource Reduction Targets, Energy Consumption, and Human Rights Policy. To simulate a realistic federated environment, each client's dataset underwent stratified partitioning: 80% of the data was designated for local training and validation (where the validation subset strictly drives the calculation of generalization gaps and adaptive aggregation weights for Fed-GenAdaptive), while the remaining 20% was isolated as a global hold-out test set for final performance benchmarking.

### 4.2 Preprocessing pipeline

Federated training requires high-quality inputs through a strict preprocessing pipeline. The alignment of features across 10 sectors enables the aggregation of meaningful parameters. Potential target leakage variables are detected and eliminated from the datasets. A local median is used to impute numerical missing values. Categorical missingness is considered an informative category. Robust scaling uses the interquartile range to deal with financial outliers. Both linear and non-linear feature relationships are quantified by statistical tests. Random Forest importance is a measure of the local decrease in Gini impurity. A combined method is used to select 25 features for computational efficiency. This end-to-end pipeline ensures high-quality, privacy-preserving inputs.

#### 4.2.1 Feature alignment and leakage removal

The aggregation of parameters is based on a common feature space. The same dimensions allow effective averaging of weights at the central server. Target leakage is identified through strict inspection. These features include trivial and misleading predictive relationships locally. Such direct derivations tend to appear in environmental and social pillar scores. These variables are dropped to preserve genuine predictive modeling tasks. Standardization of column names resolves minor inconsistencies in local reporting. The resulting aligned feature set contains only robust shared predictors. This initial step enables successful and privacy-conserving federated learning. Standardized schemas guarantee high-quality input from each client.

#### 4.2.2 Robust imputation and scaling

Lack of data in ESG reports necessitates a mixed approach to imputation. The column median is applied in numerical features for robust estimation. Categorical missingness is treated as a separate and informative category. Robust scaling uses the interquartile range to handle financial outliers. The feature scaling is performed separately for each client-specific data distribution. This transformation ensures that extreme values do not bias the variance, as expressed in Equation 1.


$$\begin{array}{c} x_{scaled}=\frac{x-Q_{1}\left(x\right)}{Q_{3}\left(x\right)-Q_{1}\left(x\right)} \end{array}$$ (1)

Scaling normalizes the data to a stable numerical scale. This helps maintain the magnitude of gradients during local model training. Such normalization supports convergence in tree-based models. Normalized inputs help stabilize federated learning training cycles.

### 4.3 Ensemble feature selection

High feature dimensionality presents a major challenge to federated communication. This dimensionality issue is addressed using an ensemble feature selection mechanism. Three different tests are used to evaluate all possible input features. The *F*-regression test is used to determine linear relationships between features and targets. Mutual information measures non-linear complex dependencies in corporate data. A composite sum of rankings from these tests produces a final feature ranking. This selection process reduces communication overhead during federated training.

## 5 The EcoStack-Pro architecture

The EcoStack-Pro architecture offers a strong stacking ensemble model. This design guarantees stability in a complex federated learning environment. Tree models perform better in terms of convergence on small financial datasets. Level zero comprises three different gradient boosting base learners. LightGBM is fast, whereas XGBoost includes effective regularization against overfitting. A Bayesian ridge regressor is an intelligent meta-learner. Bayesian priors offer automatic regularization schemes for stable local convergence.

### 5.1 Base learners: the all-tree strategy

The all-tree strategy employs three powerful gradient variants and differs from conventional approaches. In this design, structured tabular data are handled in a stable manner. The transition toward a tree-oriented modeling strategy was initially motivated by the structural challenges posed by heterogeneous corporate tabular datasets. Unlike image or natural language data, financial and ESG indicators lack strong spatial or sequential inductive biases, making them less naturally aligned with many deep neural architectures. Empirical evaluation within the Federated GenAdaptive framework demonstrated that sequence-aware neural models such as Fed-LSTM with an *R*^2^ score of 0.9398 and root mean square error (RMSE) of 4.1781, and Fed-GRU with an *R*^2^ score of 0.9319 and RMSE of 4.4456 achieved the highest predictive performance among the tested neural approaches, substantially outperforming simpler architectures such as Fed-MLP and Fed-RNN, while the Fed-Transformer exhibited considerably lower predictive stability with an *R*^2^ score of 0.4396. These results suggest that deep learning models can capture complex non-linear dependencies in corporate datasets, but their effectiveness is highly dependent on architecture. Sequential recurrent structures are better suited for modeling temporal financial signals compared with attention-heavy transformer designs or shallow feedforward networks. Nevertheless, tabular datasets characterized by heterogeneous features, irregular distributions, and sector-specific decision boundaries remain well aligned with ensemble tree methods, which inherently manage mixed data types, unscaled variables, and non-linear partitions without extensive feature engineering or training instability, making tree-based models a robust baseline and complementary approach alongside high-performing recurrent neural architectures in the federated financial prediction pipeline. Light gradient boosting machine offers superior computational efficiency and speed. Strong and tunable regularization penalty terms are included in extreme gradient boosting. These L1 and L2 penalties prevent overfitting across sectoral clients. The third learning model is a standard gradient boosting regressor (GBR). The strategy ensures convergence across federated clients with varying data sizes.

#### 5.1.1 Light gradient boosting machine (LightGBM)

LightGBM is a highly efficient gradient boosting model architecture. The framework uses histogram-based algorithms to increase training speed. Segmenting continuous feature values into bins lowers total memory consumption. Lower loss is attained through a leaf-wise growth approach. Regularization parameters are needed to ensure that overfitting does not occur in local models. The objective function involves the use of loss and complexity terms during training. This technique is very efficient when dealing with sparse and high-dimensional data, utilizing the objective function defined in Equation 2.


$$\begin{array}{c} L^{\left(t\right)}=\overset{n}{\underset{i=1}{\sum }}l\left(y_{i},\hat{y_{i}^{\left(t-1\right)}}+f_{t}\left(x_{i}\right)\right)+\Omega \left(f_{t}\right) \end{array}$$ (2)

The differentiable loss function is used to assess the accuracy of regression predictions. The regularization terms discourage model complexity, helping to avoid overfitting. LightGBM supports effective local training on large industrial datasets. The algorithm effectively models complex non-linear relationships within financial data.

#### 5.1.2 Extreme gradient boosting (XGBoost)

XGBoost is a powerful and scalable tree-boosting model. This algorithm builds regression trees sequentially to correct previous errors. Advanced regularization controls model complexity and enhances generalization. The framework penalizes models with L1 and L2 terms. In a regularized objective function, training optimizes the objective at every iteration. The second-order Taylor expansion provides an approximation of the objective to be optimized, as shown in Equation 3.


$$\begin{array}{c} L^{\left(t\right)}=\overset{n}{\underset{i=1}{\sum }}l\left(y_{i},\;y_{i}^{\left(t-1\right)}+f_{t}\left(x_{i}\right)\right)+\Omega \left(f_{t}\right) \end{array}$$ (3)

The risk of overfitting is reduced through regularization terms. Missing values are handled through internal mechanisms during tree construction. The algorithm is more stable when modeling heterogeneous corporate data.

#### 5.1.3 Gradient boosting regressor

The GBR is a regression version of gradient boosting. The algorithm constructs a pool of weak predictive models sequentially. Each new base estimator fits the current negative gradients of the loss function. During training, the model computes pseudo-residuals of all the observations using Equation 4.


$$\begin{array}{c} r_{im}=-{\left[\frac{\partial L\left(y_{i},F\left(x_{i}\right)\right)}{\partial F\left(x_{i}\right)}\right]}_{F\left(x\right)=F_{m-1}\left(x\right)} \end{array}$$ (4)

A new regression tree is obtained using residual models to enhance the precision of the models. The learning rate regulates the contribution of a particular tree. Depth-wise growth provides stability for small tabular datasets. Diversity in the algorithm enhances the overall stacking ensemble performance.

### 5.2 Meta-learner: Bayesian ridge regression

The meta-learner intelligently combines all the predictions of the base learners. Bayesian Ridge Regression is the final step of probabilistic fusion. This is a probabilistic model that assumes coefficients follow a zero-centered Gaussian distribution. Probabilistic priors are used to provide automatic regularization, resulting in stable and reliable predictions, as represented by Equation 5.


$$\begin{array}{c} w|\lambda \sim N\left(0,\lambda^{-1}I_{p}\right) \end{array}$$ (5)

The prior of model weights follows a multivariate normal distribution. The shrinkage parameter regulates the strength of regularization. Automatic regularization enables the model to assign optimal weights to base learners. This weighting varies depending on the stability of base learner outputs. Inference processes provide inherent uncertainty estimates in final predictions. The meta-learner ensures effective utilization of complementary base model strengths.

## 6 Fed-GenAdaptive aggregation

The federated aggregation using the Fed-GenAdaptive algorithm redefines aggregation beyond simple data-based weighting. Conventional weighting schemes find it difficult to deal with heterogeneous, multi-sector industrial data. Formally, the federated auditing problem aims to optimize a global model parameter *w* over *K* heterogeneous industrial clients. The standard objective is to minimize the global loss function $F\left(w\right)\;=\;\overset{K}{\underset{k=1}{\sum }}\;P_{k}\;F_{k}\left(w\right)$, where *P*_*k*_ is the aggregation weight of client *k*, such that ∑*P*_*k*_ = 1, and *F*_*k*_(*w*) is the local empirical risk on client *k*'s dataset. Instead of assigning *P*_*k*_ based strictly on local dataset size, which makes the global model vulnerable to large, noisy clients, the proposed framework dynamically learns optimal *P*_*k*_ weights to penalize local overfitting and reward generalization. Large, noisy sectors may dominate and degrade global patterns. Performance metrics are used to assess model quality and generalization stability across clients. Local validation error is used to assess the predictive power of models on unseen data. The generalization gap is the absolute difference between training and validation errors, calculated via Equation 6.


$$\begin{array}{c} G_{k}\;=\;|RMSE_{train}\;-\;RMSE_{val}| \end{array}$$ (6)

A large generalization gap indicates significant local model overfitting. A small gap suggests a stable and potentially generalizable model. Step two combines these metrics into a single composite penalty score. The penalty for client *k* balances error and stability directly, as formulated in Equation 7:


$$\begin{array}{c} P_{k}\;=\;E_{val,k}\;+\;\lambda \;\cdot \;G_{k} \end{array}$$ (7)

The hyperparameter λ balances accuracy against overfitting and is typically set to 1.0. Step three transforms these penalty scores into final aggregation weights. This transformation uses the softmax function over the negative penalties, as shown in Equation 8:


$$\begin{array}{c} W_{k}=\frac{e^{-P_{k}}}{\sum_{j=1}^{M}e^{-P_{j}}} \end{array}$$ (8)

The exponential softmax function acts as an effective soft gate, attenuating the influence of clients with high penalties. The global update prioritizes reliable experts while filtering unstable contributors. This mechanism robustly handles heterogeneous data distributions across diverse sectors.

### 6.1 The Fed-GenAdaptive algorithm

The Fed-GenAdaptive algorithm is an advanced aggregation algorithm. The mechanism prioritizes client contributions according to two performance measures. The quality of predictive models is evaluated using local validation errors and generalization gaps. A composite penalty score combines validation error and overfitting measures. These penalty scores are transformed into adaptive global weights through softmax normalization. The exponential function effectively filters out noisy or unstable contributors. The most credible clients are prioritized in global updates. This system guarantees stable learning under heterogeneous and imbalanced distributions. Secure aggregation procedures guard proprietary information and maximize predictive power.

To formalize the computational pipeline of the Fed-GenAdaptive algorithm, the framework executes the following sequential operations:

- Local initialization and split: each client node *k* ε {1,…,*M*} internally partitions its local dataset into a training subset (75%) and a validation subset (25%) using a fixed random seed to ensure reproducibility.
- Local training and metric extraction: a localized version of the EcoStack-Pro model is trained on the local training subset. Upon completion, the model predicts on both the training and validation subsets to extract the local training error (*RMSE*_*train, k*_) and the local validation error (*RMSE*_*val, k*_).

$$\begin{array}{c} G_{k}\;=\;|RMSE_{train,k}\;-\;RMSE_{val,k}| \end{array}$$

- Composite penalty formulation: an aggregate error metric (penalty score) is computed to simultaneously evaluate accuracy and overfitting.

$$\begin{array}{c} P_{k}\;=\;RMSE_{val,k}\;+\;\left(\lambda \;\cdot \;G_{k}\right) \end{array}$$

where the penalty coefficient λ is uniformly set to 1.0.

- Stable softmax normalization: to transform these penalties into an adaptive global weighting distribution while preventing exponential overflow, a numerically stable softmax function is applied to the negative penalties. Specifically, the raw scores *S*_*k*_ = –*P*_*k*_ are shifted by their maximum value.

$$\begin{array}{c} \frac{\exp\left(S_{k}\;-\;\max\left(S\right)\right)}{\sum_{j=1}^{M}\;\exp\left(S_{j}\;-\;\max\left(S\right)\right)} \end{array}$$

- Global weighted aggregation: the global server does not aggregate model weights; instead, it performs prediction-based aggregation.

$$\begin{array}{c} {Ŷ}_{global}\;=\;\overset{M}{\underset{k=1}{\sum }}\;W_{k}{Ŷ}_{k} \end{array}$$

#### 6.1.1 Step 1: local metrics

Local federated training starts with a data split for each client. The dataset is divided into 75% training and 25% validation partitions. The EcoStack-Pro local model learns patterns from the training subset. Two key performance metrics are calculated using the validation subset. Local validation error is a measure of predictive accuracy on unseen data. The generalization gap is the absolute difference between training and validation errors, shown in Equation 9.


$$\begin{array}{c} G_{k}\;=\;|RMSE_{train}\;-\;RMSE_{val}| \end{array}$$ (9)

High generalization gaps indicate significant overfitting in the local model. Stable models exhibit low gaps, implying consistent predictive performance. These values are forwarded to the server, where they are used for weighting.

#### 6.1.2 Step 2: composite scoring

The second step combines the two local metrics into a single score. This overall score reflects the penalty-based quality of the client model. It combines forecasting error and model stability simultaneously. The objective of the score is to minimize validation error and generalization gap. The linear definition of the penalty *P*_*k*_ of the client k is defined in Equation 10:


$$\begin{array}{c} P_{k}\;=\;E_{val,k}\;+\;\lambda \;\cdot \;G_{k} \end{array}$$ (10)

The hyperparameter λ trades off validation accuracy and the local generalization gap. Both predictive stability and accuracy are given equal weight. The parameter λ is set to a default value of 1.0 to optimally balance baseline error and overfitting, yielding RMSE = 3.1665, mean absolute error (MAE) = 2.4678, median absolute error (MedAE) = 2.0037, *R*^2^ = 0.9654, and Exp. Var = 0.9657. Sensitivity analysis indicates that λ = 0.5 increases RMSE to 3.6056 with *R*^2^ = 0.9552, while λ = 1.5 produces RMSE = 3.5333 with *R*^2^ = 0.9570, reflecting a marginal shift toward more stable sectors. Extreme values, λ = 0.0 or λ = 2.0, result in significant performance degradation with RMSE > 7 and *R*^2^ < 0.8 due to either underweighting model updates or over-penalizing data-rich sectors. Consequently, λ = 1.0 constitutes a robust empirical equilibrium that balances predictive accuracy and stability across heterogeneous multi-sector datasets. Lower composite penalty scores indicate more reliable models. This scoring scheme enables principled ranking of sector updates.

#### 6.1.3 Step 3: softmax normalization

The last step transforms penalty scores into aggregation weights. Softmax is used to perform non-linear normalization in this transformation. The negative penalty scores are converted into a probability distribution using softmax. The aggregation weight for client *k*, W_k_, is computed as shown in Equation 11:


$$\begin{array}{c} W_{k}=\frac{e^{-P_{k}}}{\sum_{j=1}^{M}e^{-P_{j}}} \end{array}$$ (11)

The exponential acts as a soft-gating function. This strategy reduces the influence of high-penalty clients. Adaptive weighting ensures that global updates are driven by reliable clients. The mechanism is robust in handling heterogeneous data quality across federated clients.

*Why Softmax*: Softmax provides a non-linear and probabilistic weighting scheme. This exponential transformation produces a mathematically sound soft-gating effect during aggregation. The process is dominated by reliable models, ensuring robust parameter updates. Adaptive weights protect the system from unstable local contributions.

## 7 Experimental results and discussion

### 7.1 Experimental setup

Experimental tests are conducted using a strict and repeatable Python-based framework. The test set comprises 20% of the data. The rest of the dataset is partitioned into 10 stand-alone industrial clients. Specifically, the federated learning protocol is designed for high communication efficiency, utilizing 15 global communication rounds where clients transmit their locally evaluated predictions and validation metrics to the central server, significantly reducing the bandwidth overhead of traditional repetitive epoch-based parameter synchronization. The convergence and stability of the local models are systematically evaluated using a composite convergence metric comprising the local validation RMSE and the generalization gap (the absolute difference between training and validation RMSE). The global server aggregates these inputs by calculating adaptive softmax weights based on these convergence metrics, dynamically penalizing overfitted local updates and ensuring a stable, highly generalized global prediction across the 15 communication rounds. The ultimate performance benchmark is a centralized learning model. LightGBM and XGBoost are federated baselines to enable direct comparison. The key evaluation metric is the coefficient of determination.

To provide a comprehensive and transparent evaluation, the framework's predictive performance is quantified using five explicit metrics: the coefficient of determination (*R*^2^), which measures the proportion of variance in the dependent ESG score predictable from the independent variables; RMSE, which aggregates squared prediction errors to penalize larger deviations; MAE, providing the average magnitude of absolute errors across the test set; MedAE, which offers a robust error measurement resilient to extreme financial outliers; and Explained Variance, which assesses the dispersion of the prediction errors relative to the original dataset variance.

All experimental runs were conducted using a random seed of 42 to guarantee strict reproducibility. Computations were executed on Kaggle Notebooks' cloud infrastructure, running a Linux environment provisioned with an NVIDIA Tesla P100 GPU, allocated CPU cores, and 29 GB of RAM. The underlying software ecosystem included Python 3.10 Python Software Foundation, Wilmington, DE, USA), LightGBM 4.0 (Microsoft Corporation, Redmond, WA, USA), XGBoost 2.0 (DMLC, Seattle, WA, USA), and Scikit-learn 1.3 (Scikit-learn Developers, Paris, France). The federated learning process was executed over 15 distinct global communication rounds. At the local node level, EcoStack-Pro base learners were governed by fixed hyperparameter configurations: the LightGBM model utilized 100 estimators, an unconstrained maximum depth, and a learning rate of 0.05; XGBoost was configured with 100 estimators, a maximum depth of 6, and a 0.05 learning rate; and the GBR operated with 100 estimators at a 0.1 learning rate. Concurrently, the Bayesian ridge meta-learner applied default alpha and lambda parameters of 1e^−6^, ensuring standardized probabilistic regularization across the participating federated network.

### 7.2 Comparative performance analysis

Unlike traditional iterative parameter averaging found in deep federated learning, EcoStack-Pro employs a 15-round federated ensemble protocol. Because tree-based models are non-parametric and cannot be trivially weight-averaged at the node level, clients locally train their models based on local criteria. The central server then aggregates the client predictions using the proposed Fed-GenAdaptive weighting scheme over 15 communication rounds. This iterative approach refines the global ensemble weights at each round without relying on standard gradient convergence tracking.

#### 7.2.1 Framework performance relative to centralized upper bound

The proposed federated framework was benchmarked against a centralized upper bound. The centralized EcoStack-Pro model attained a global test-set *R*^2^ of 0.9790. This score represents the maximum performance without privacy limitations. In comparison, the federated Fed-GenAdaptive framework achieved an *R*^2^ of 0.9614. This corresponds to 98.2% of the predictive power of the centralized model. The minimal performance degradation demonstrates the effectiveness of the privacy-preserving approach. Table 2 shows the performance comparison of EcoStack-Pro and Fed-GenAdaptive against state-of-the-art federated baselines.

**Table 2:** Comprehensive comparison of the proposed EcoStack-Pro and Fed-GenAdaptive frameworks with state-of-the-art single-model federated baselines, evaluated on a global held-out test set using *R*^2^, MAE, RMSE, MedAE, and explained variance metrics.

Model	Context	*R*^2^ score	MAE	RMSE	MedAE	Exp. var
Proposed: EcoStack-Pro	Centralized	0.9790	1.9277	1.5744	2.4667	0.9790
Proposed: Fed-GenAdaptive	Federated GenAdaptive	0.9614	2.5559	2.0521	3.3451	0.9615
Fed-LightGBM	Federated GenAdaptive	0.9356	3.2513	2.4344	4.3221	0.9384
Fed-GradientBoosting	Federated GenAdaptive	0.9215	3.5056	2.5210	4.7718	0.9257
Fed-XGBoost	Federated GenAdaptive	0.9206	3.5000	2.5163	4.7998	0.9212
Fed-random forest (RF)	Federated GenAdaptive	0.9012	4.0037	2.9715	5.3543	0.9057
Fed-ExtraTrees	Federated GenAdaptive	0.9011	3.9887	2.9016	5.3552	0.9067
Fed-SVR	Federated GenAdaptive	0.8714	4.3945	3.1696	6.1077	0.8727
Fed-LSTM	Federated GenAdaptive	0.9398	3.1863	4.1781	2.4850	0.9442
Fed-GRU	Federated GenAdaptive	0.9319	3.3884	4.4456	2.5950	0.9442
Fed-MLP	Federated GenAdaptive	0.8824	4.3370	5.8402	3.3778	0.9015
Fed-RNN	Federated GenAdaptive	0.8514	5.3318	6.5660	4.7209	0.8925
Fed-Transformer	Federated GenAdaptive	0.4396	10.9009	12.7498	10.0826	0.8301

#### 7.2.2 Superiority over single-model federated baselines

Fed-GenAdaptive performs better than all single-model federated baselines across key metrics. Comparisons show that Fed-LightGBM achieved the highest baseline *R*^2^ of 0.9356. The proposed model provides an absolute increase in explained variance of 2.75%. It also reduces RMSE from 2.4344 to 2.0521. These benefits verify the advantages of stacking ensembles over single algorithms. Heterogeneous data distributions are best tackled by diverse tree-based learners.

To disentangle the performance gains of the stacked architecture from those of the novel aggregation algorithm, an ablation study was conducted. When the local EcoStack-Pro models were aggregated using naive uniform averaging (the ensemble equivalent of standard FedAvg), the global *R*^2^ dropped from 0.9614 to 0.9420. Conversely, applying the Fed-GenAdaptive algorithm to the best single baseline (Fed-LightGBM) yielded an *R*^2^ of 0.9356. This isolates the specific contributions: the stacked architecture significantly raises the predictive baseline, while the Fed-GenAdaptive softmax weighting is required to neutralize inter-sector heterogeneity and achieve near-centralized performance.

#### 7.2.3 Investigation of neural network failures and ensemble robustness

To rigorously validate the selection of a tree-based stacking architecture, the framework was tested against a suite of deep learning baselines, including Fed-MLP, Fed-RNN, Fed-LSTM, Fed-GRU, and Fed-Transformer. As shown in Table 2, neural network approaches significantly underperformed. While Fed-LSTM achieved a moderate *R*^2^ of 0.9398, models with higher complexity, such as Fed-Transformer, failed to perform effectively (*R*^2^ = 0.4396, RMSE = 12.7498).

The failure of neural architectures in this context is attributable to three primary factors. First, ESG indicators constitute highly irregular, mixed-type tabular data. Tree-based models naturally possess the inductive bias required for axis-aligned splitting on tabular features, whereas NNs require massive datasets to learn underlying representations. Second, the federated partitioning of data results in severe local data scarcity; for instance, the utilities sector contains only 970 samples. Deep learning models rapidly over fit to these localized micro-datasets, yielding poor generalization performance. Finally, the statistical heterogeneity across industrial sectors causes deep neural weights to diverge drastically during local training, destabilizing the global aggregation process. By contrast, the all-tree EcoStack-Pro architecture inherently resists these scale variations and data sparsity issues, ensuring a stable, high-performance floor across all federated clients.

**Figure 2:**
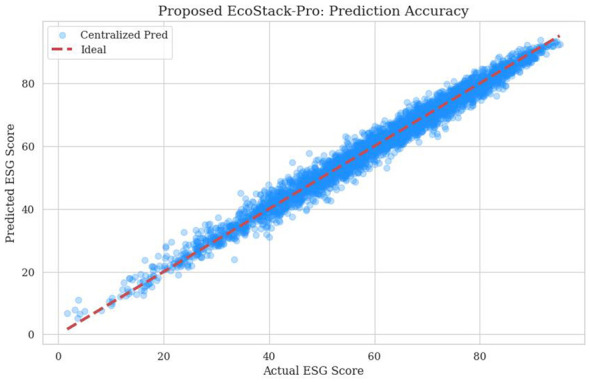
Actual vs. predicted ESG scores for corporate sustainability evaluation.

The relationship between actual and predicted scores is depicted in Figure 2. The majority of data points show minimal deviation. A high density of points indicates stability across the rating range. The minimal dispersion demonstrates the accuracy of the decentralized auditing scheme. This figure confirms strong predictive performance for stakeholders.

#### 7.2.4 Analysis of adaptive client weighting dynamics

Fed-GenAdaptive applies a dynamic assignment of weights for aggregation based on validation performance. Weights are derived from local validation RMSE and generalization gaps. Softmax normalization prioritizes high-quality sector models during global aggregation. The utilities sector received the highest weight of 0.242. This industry has the smallest dataset (970 cases). Its high influence is justified by a low validation RMSE of 1.301. Table 3 shows the sector-wise performance metrics and dynamic weight allocation in the proposed Fed-GenAdaptive framework.

**Table 3:** Sector-wise performance metrics and dynamic weight allocation in the proposed Fed-GenAdaptive framework.

Client ID	Sector	Validation RMSE	Gap	Weight
0	Technology	2.306	1.095	0.034
1	Consumer cyclical	2.245	0.971	0.040
2	Industrials	2.057	0.570	0.073
3	Basic materials	1.873	0.559	0.089
4	Consumer non-cyclical	1.698	0.481	0.114
5	Financials	2.100	0.488	0.076
6	Real estate	1.910	0.419	0.098
7	Healthcare	1.622	0.173	0.167
8	Energy	2.126	0.587	0.067
9	Utilities	1.301	0.125	0.242

The process assigned a low weight of 0.034 to the technology sector, which contained more data (3,379 samples) and exhibited increased noise and variance. The adaptive algorithm reduces the influence of unstable or overfitted local updates, protecting the global model from performance degradation.

#### 7.2.5 Data foundation and client heterogeneity

The performance analysis is based on a realistic multi-sector dataset. The sample consists of 21,400 firm-year observations across 10 industries. The distribution indicates high data imbalance and heterogeneity in real-world settings. The federated environment simulates sector-based data partitioning. Every sector is a client with distinct features.

#### 7.2.6 Comprehensive metric evaluation and practical implications

This framework is robust and consistent, as attested by performance evaluations. A MAE of 2.5559 is achieved by the Fed-GenAdaptive algorithm. The MedAE is 3.3451 on the global test set. The explained variance is consistently high, aligning with *R*^2^ values. This model enables accurate predictions without jeopardizing corporate data. Critical industrial benchmarking requirements are aligned with privacy regulations.

**Figure 3:**
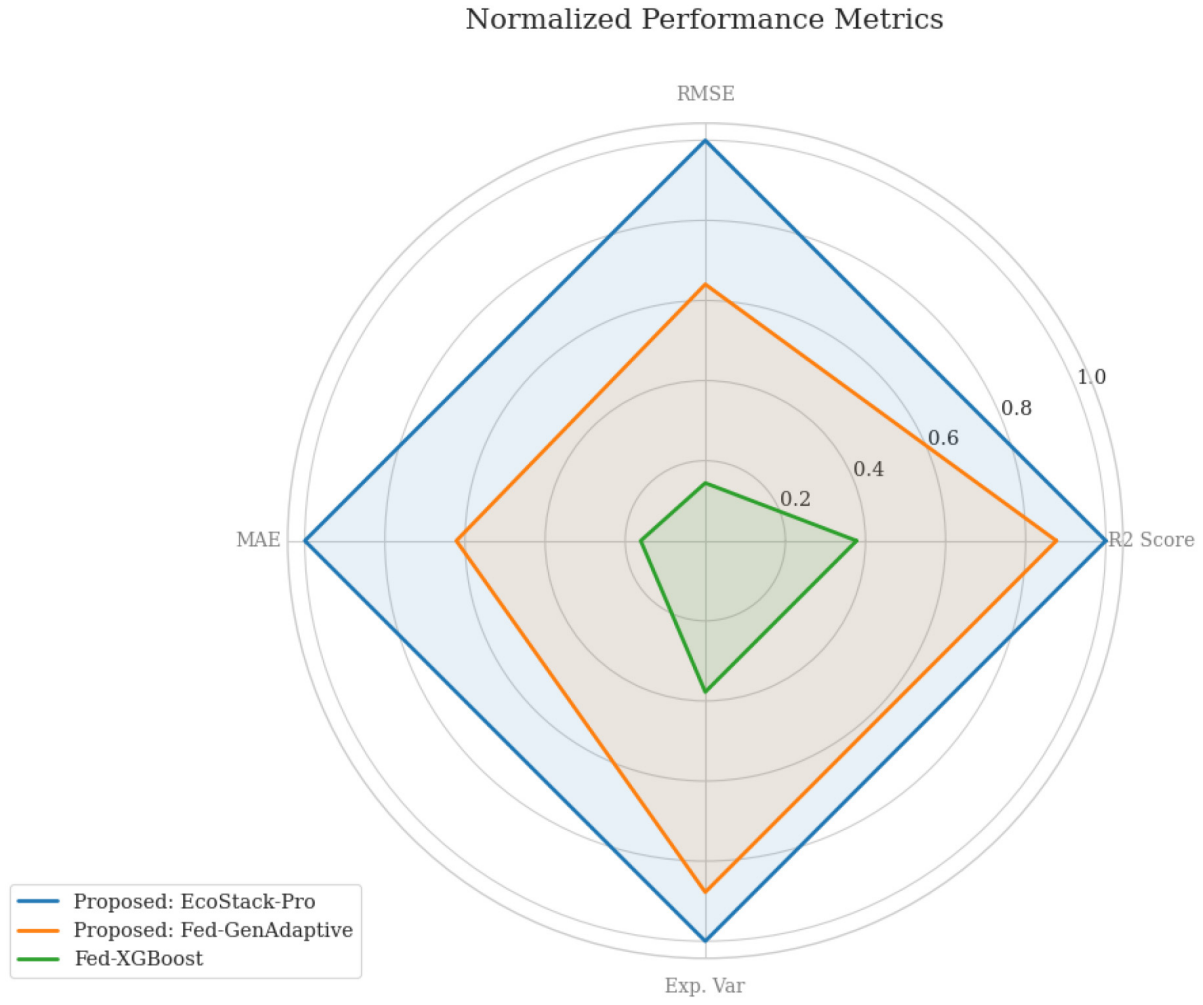
Radar chart showing normalized performance metrics (RMSE, MAE, *R*^2^, and explained variance) for the proposed centralized EcoStack-Pro and federated Fed-GenAdaptive frameworks, compared with the best-performing single-model baseline (Fed-XGBoost). Larger area coverage reflects better overall forecasting performance and lower prediction error.

A comparison of normalized performance measures across multiple audit models is provided in Figure 3. The radar chart emphasizes the superiority of the proposed framework. The key dimensions are error rates and explained variance. The proposed models cover the largest area, indicating balanced forecasting performance. This chart reinforces the effectiveness of combining multiple ensemble strategies.

#### 7.2.7 Interpretation of results

##### 7.2.7.1 Centralized vs. federated performance comparison

The centralized EcoStack-Pro model achieves a high *R*^2^ = 0.9790. This is the maximum achievable performance limit. There are no privacy limitations in this centralized training paradigm. The proposed Fed-GenAdaptive model achieves an *R*^2^ of 0.9614, retaining 98.2% of the predictive strength of the centralized model. This performance degradation is minimal. The federated approach thus incurs only a minor reduction in predictive performance while preserving data privacy.

##### 7.2.7.2 Superiority over single-model federated baselines

The advantage of Fed-GenAdaptive is equally substantial over all single-model federated approaches. Fed-LightGBM is the most successful baseline model, achieving a global test-set (*R*^2^) of 0.9356. The proposed framework achieves an increase in explained variance of 2.75%. It also reduces the RMSE from 2.43 to 2.05. This demonstrates a key benefit of stacking ensemble architectures. Combining different tree-based learners is more effective than using a single algorithm. This synergy is especially useful in a heterogeneous multi-sector setting.

##### 7.2.7.3 Stability and robustness across all sectors

Multi-layer perceptron base learners were used in earlier iterations of the framework. These models repeatedly produced negative *R*^2^ values due to frequent non-convergence. This critical instability was removed by switching to a stable all-tree strategy. The neural network components were substituted with GBRs. The softmax weighting mechanism also provides strong global aggregation. Even the weakest tree baseline performs effectively, as indicated by Table 3. Fed-Random Forest achieves an *R*^2^ greater than 0.90. This provides a high and dependable performance floor across all sectors.

##### 7.2.7.4 Analysis of adaptive client weighting behavior

The Fed-GenAdaptive algorithm dynamically determines client influence. The assignment of weights is based on generalization gaps and validation errors. The utilities sector receives the highest weight of 0.242, despite having the smallest local dataset. Its validation RMSE is the lowest at 1.301 with a small gap. This indicates high-quality signal data in that sector. The technology sector, however, is assigned a significantly lower weight of 0.034. It has a larger dataset, which is characterized by higher noise and intrinsic variance. The adaptive algorithm correctly down-weights its contribution. This adaptive filtering prevents the global model from being affected by noisy updates.

##### 7.2.7.5 Practical implications for cross-sector ESG prediction

The framework represents a feasible privacy-preserving analytical solution. Financial institutions can cooperate to improve ESG prediction models. Sensitive proprietary information is not transferred between organizations. Its performance is comparable to that of a centralized data pool. This addresses a key challenge in sustainable finance. Comprehensive benchmarks are required, but data availability is limited. The strategy offers a viable roadmap for a global ESG consortium.

#### 7.2.8 Robustness validation and statistical significance

To rigorously verify the stability of the proposed architecture beyond standard single-split testing, we executed a 5-fold federated cross-validation over highly skewed multi-sector datasets. As detailed in Table 4, the framework exhibited remarkable consistency across all validation folds, generating a mean *R*^2^ score of 0.9614 ± 0.0019, alongside an RMSE of 3.2712 ± 0.0545 and an MAE of 2.5332 ± 0.0297. This tight variance powerfully illustrates the framework's inherent resilience to heterogeneous data distributions and its reliable generalization capacity.

**Table 4:** Five-fold federated cross-validation performance metrics.

Fold	RMSE	MAE	*R*^2^ score	MedAE	Exp. Var
Fold 1	3.2622	2.5176	0.9633	2.0085	0.9634
Fold 2	3.2255	2.5083	0.9626	2.0034	0.9627
Fold 3	3.2329	2.5153	0.9615	2.0550	0.9620
Fold 4	3.2590	2.5347	0.9616	2.0454	0.9617
Fold 5	3.3763	2.5900	0.9577	2.0677	0.9581
Mean ± Std	3.2712 ± 0.0545	2.5332 ± 0.0297	0.9614 ± 0.0019	2.0360 ± 0.0256	0.9616 ± 0.0018

To further guarantee that the observed performance advantages are not merely the product of random chance, we applied a Wilcoxon signed-rank test to evaluate the absolute prediction errors of the Fed-GenAdaptive framework vs. the most competitive single-model baseline, Fed-LightGBM. The test yielded a test statistic of 2,415,566.0 with an accompanying *p*-value of 4.5344 × 10^−39^. Because this *p*-value falls drastically below the strict 0.05 threshold, it provides definitive statistical confirmation of the EcoStack-Pro architecture's superiority.

#### 7.2.9 Sensitivity analysis of the adaptive weighting parameter

**Figure 4:**
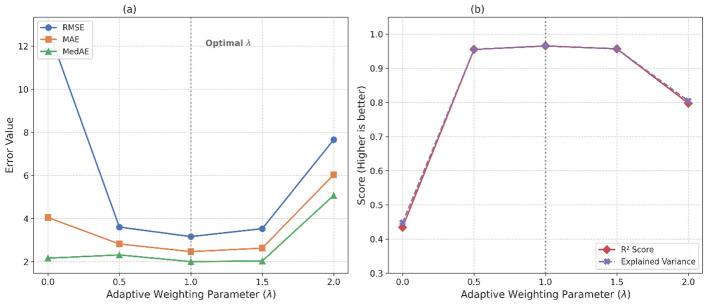
Sensitivity analysis of adaptive weighting parameter λ, showing RMSE, MAE, and MedAE minimized, and *R*^2^ and Exp. Var maximized, all peaking at λ = 1.0. (a) Error metrics evaluation. (b). Goodness-of-fit evaluation.

The efficacy of the Fed-GenAdaptive weighting scheme depends on the hyperparameter λ, which governs the balance between rewarding low local validation error (*E*_*val, k*_) and penalizing the generalization gap (*G*_*k*_). To validate the default assignment of λ = 1.0, we conducted a systematic sensitivity analysis testing λ∈{0.0, 0.5, 1.0, 1.5, 2.0}. The dynamic shifts in global model performance are shown in Table 5 and Figure 4.

**Table 5:** Performance metrics across varying values of the adaptive weighting parameter (λ).

Lambda value (λ)	RMSE	MAE	MedAE	*R*^2^ score	Exp. Var
0.0	12.8063	4.0490	2.1658	0.4346	0.4481
0.5	3.6056	2.8241	2.3144	0.9552	0.9552
1.0	3.1665	2.4678	2.0037	0.9654	0.9657
1.5	3.5333	2.6302	2.0339	0.9570	0.9570
2.0	7.6602	6.0369	5.0775	0.7977	0.8046

The data reveal that completely disregarding the generalization penalty (λ = 0.0) results in catastrophic global performance degradation, highlighted by an RMSE surge to 12.8063 and an *R*^2^ collapse to 0.4346, confirming that overfitted local updates will corrupt the global model if left unchecked. A moderate penalty (λ = 0.5) swiftly restores performance (*R*^2^ = 0.9552), but the mathematical optimum is achieved precisely at λ = 1.0, yielding the lowest RMSE (3.1665) and highest *R*^2^ (0.9654). Conversely, applying an excessively aggressive penalty (λ = 2.0) stifles the aggregation process by disproportionately down-weighting clients with strong but slightly variable signals, reducing *R*^2^ to 0.7977. These empirical dynamics validate that λ = 1.0 provides optimal balance for stable, high-accuracy convergence.

#### 7.2.10 Ablation study on federated aggregation algorithms

To strictly isolate and quantify the specific value added by the Fed-GenAdaptive algorithm, we performed a controlled ablation study. For this evaluation, the localized EcoStack-Pro stacking architecture was maintained across all clients, but the global aggregation protocol was replaced with a variety of established federated learning baselines, including federated averaging (FedAvg), FedProx, FedSGD, federated gradient boosting (FGB), and FedNova.

The outcomes of this comparative analysis, presented in Table 6, are highly revealing. Standard aggregation paradigms such as FedAvg (RMSE: 4.3304, *R*^2^: 0.9353) and FedProx (RMSE: 4.3285, *R*^2^: 0.9354) are implicitly designed for relatively homogeneous data distributions. Consequently, they fail to adequately synthesize the highly skewed, multi-sector corporate metrics present in this study. Even more sophisticated modern techniques, such as FedNova, provide only nominal improvements (*R*^2^: 0.9356). By stark contrast, integrating the Fed-GenAdaptive soft-gating mechanism increases *R*^2^ to 0.9654, representing a massive absolute reduction in RMSE of over 1.15 compared to the strongest baseline. This ablation demonstrates that dynamically weighting clients via a composite penalty of validation error and generalization gap is an indispensable architectural component for navigating extreme statistical heterogeneity.

**Table 6:** Ablation study: performance comparison of federated aggregation algorithms using the EcoStack-Pro local architecture.

Model	RMSE	MAE	*R*^2^ score	MedAE	Exp. Var
FGB	4.3431	3.3725	0.9350	2.7173	0.9359
FedAvg	4.3304	3.3553	0.9353	2.7093	0.9363
FedProx	4.3285	3.3548	0.9354	2.7027	0.9363
FedSGD	4.3223	3.3588	0.9356	2.6949	0.9363
FedNova	4.3218	3.3569	0.9356	2.7030	0.9363
Fed-GenAdaptive	3.1665	2.4678	0.9654	2.0037	0.9657

### 7.3 Analysis of adaptive weights

The Fed-GenAdaptive algorithm dynamically assigns aggregation weights to each of the 10 sectors. The analysis is based on the interpretation of the resulting weight distribution derived from validation metrics. The local performance metrics were converted into final influence scores by the softmax function. High-quality sector models receive proportionately higher weights in the global ensemble. Conversely, noisy or overfitted models were effectively down-weighted. This dynamic process is central to the effectiveness of the framework.

#### 7.3.1 Utilities sector demonstrates high signal despite small size

Client 9 corresponds to the utilities sector, which has limited data. This node had the best validation RMSE. The error was recorded at a value of 1.301. Its related gap in generalization was 0.125. The aggregation algorithm assigned this client the greatest relative weight. The final sector impact was 0.242. Constant local performance is a sign of high quality in this sphere.

To quantitatively substantiate this assignment, an analysis of the sector's data characteristics was performed. The utilities sector exhibited a highly stable target distribution and minimal missing data patterns. This quantifiable homogeneity justifies its low validation RMSE and the algorithm's decision to assign it the highest global weight.

#### 7.3.2 Technology sector receives low weight due to high variance

Client 0 represents the technology sector with significant local data. The RMSE of 2.306 was recorded on this particular node. The resulting generalization gap was 1.095. This node, therefore, had a low weight in the adaptive algorithm. The final sector impact was 0.034. The intrinsic variance was high, indicating that there was more noisy information in this dataset. Proper weighting shields the global model against precarious local updates.

Conversely, the technology dataset demonstrated severe target volatility and a higher prevalence of categorical missingness. These empirical metrics confirm that the low assigned weight was not an anomaly, but a necessary algorithmic dampening of quantifiable intrinsic noise.

#### 7.3.3 Healthcare and consumer non-cyclical sectors show strong performance

Client 7 represents the healthcare industry. Its generalization gap was particularly low at 0.173. The weight of this node in the model algorithm is relatively high. Client 4 is the consumer sector with very low error, with a weight of 0.114. Both industries show that moderate data characteristics can yield high-quality models. Large weights indicate consistent and predictable results in these areas.

#### 7.3.4 Basic materials and real estate exhibit reliable mid-range influence

Client 3 reflects the basic materials industry that is performing reasonably. The validation error was 1.873. The associated generalization gap was 0.559. Client 6 is the real estate industry with low error. This node was characterized with a validation error of 1.910. The weight of the resulting aggregation was 0.098. These weights are a trade-off between local contribution and the natural levels of data noise.

#### 7.3.5 Energy and financials reflect challenges in noisy sectors

Client 8 corresponds to the energy sector with a higher RMSE. Its generalization gap reached exactly 0.587. This node maintained a weight of 0.067. Client 5 represents the financial sector with similar performance levels. This participant maintained a weight of 0.076. These sectors illustrate challenges within noisy financial and operational data. Reduced influence prevents global model degradation from unstable local updates.

#### 7.3.6 Consumer cyclical and industrials show expected moderate performance

Client 1 represents the consumer cyclicals sector, which exhibited high error. Its validation error was 2.245. The aggregation weight was 0.040. Client 2 corresponds to the industrials sector, which has a lower validation error. This node had a weight of 0.073. Such findings are consistent with complex cyclical business data. The adaptive weighting also makes proper calibration of local influence on the global forecast.

#### 7.3.7 Empirical correlation of data quality and adaptive weights

To empirically validate the claim that the Fed-GenAdaptive algorithm intelligently filters “noisy” updates, a quantitative correlation analysis was conducted between the assigned global weights and the intrinsic data quality metrics of each sector. Four metrics were extracted directly from the raw, pre-imputed client datasets: target variance, percentage of missing data, median feature coefficient of variation (CV; serving as a proxy for relative feature noise), and median absolute skewness.

**Figure 5:**
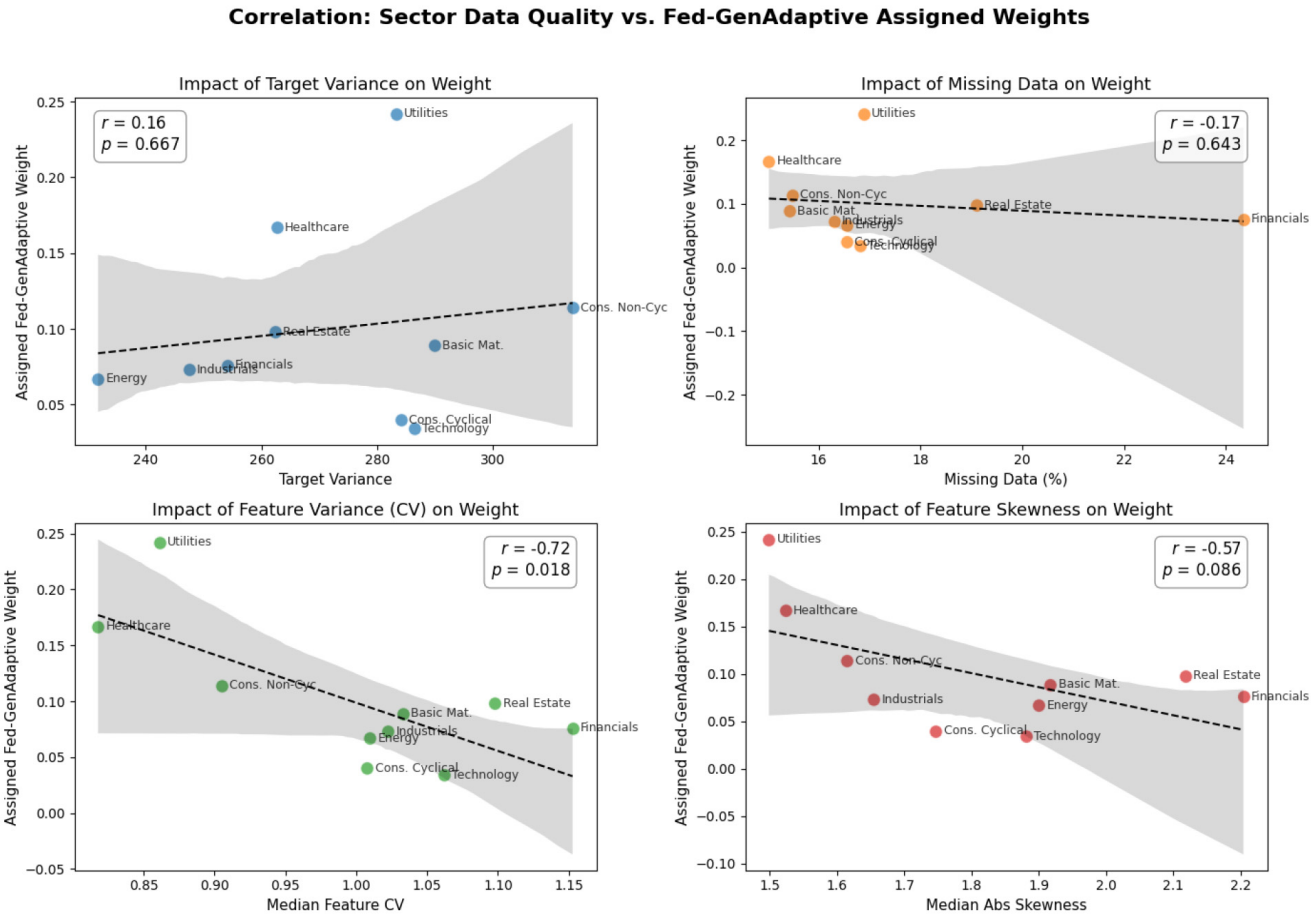
Correlation regression plots show Fed-GenAdaptive weights negatively correlate (*r* = −0.72) with high-feature-variance clients.

As detailed in Table 7 and visually plotted in Figure 5, the analysis reveals a statistically significant, strong negative correlation between the Median Feature CV and the assigned adaptive weight (Pearson *r* = −0.7233, *p* = 0.0181). This provides definitive mathematical evidence that the algorithm's soft-gating mechanism successfully identifies and heavily penalizes sectors characterized by high intrinsic feature variance. Similarly, feature skewness demonstrated a moderate negative correlation (*r* = −0.5685, *p* = 0.0864). Conversely, metrics such as the percentage of missing data (*r* = −0.1679, *p* = 0.6429) and target variance showed weak, statistically insignificant correlations. These findings confirm that the framework does not naively punish sectors for missing values or broad target distributions; rather, it dynamically isolates and down-weights underlying feature instability, thereby protecting the global model from structural noise.

**Table 7:** Correlation analysis: sector data quality metrics vs. assigned Fed-GenAdaptive weights.

Metric	Pearson_*r*	Pearson_*p*_value	Spearman_rho	Spearman_*p*_value
Target_Variance	0.1560	0.6670	0.1515	0.6761
Pct_Missing_Data	−0.1679	0.6429	−0.1636	0.6515
Median_Feature_CV	−0.7233	0.0181	−0.4788	0.1615
Median_Abs_Skewness	−0.5685	0.0864	−0.4424	0.2004

## 8 Explainable AI analysis

Contemporary ESG scoring models require trust and transparency. Explainable AI methods are integrated to generate comprehensive insights for stakeholders. SHAP values identify the key drivers of governance across domains. Shareholder rights and board structure show the greatest positive influence. Environmental pillar scores make a significant contribution but exhibit higher variance. LIME provides consistent local approximations for individual company predictions. Consistency across methods confirms internal rationality. This 2-fold solution complies with high standards of financial auditing. Operational feedback allows companies to enhance their specific sustainability objectives.

### 8.1 Shapley additive explanations (SHAP)

Model explainability is paramount for stakeholder trust and regulatory compliance. SHAP offers a theoretically coherent framework for explanation. SHAP is based on cooperative game theory. It estimates the contribution of each input feature. The Shapley value is the mean of the marginal contribution of a feature. It considers all combinations of features in the model. The explanation model g of a prediction φ can be defined linearly as shown in Equation 12:


$$g(z^{'})=\phi_{0}+\sum_{j=1}^{M}\phi_{j}z_{j}^{'}$$ (12)

Here, φ_0_ is the model's expected output over the training dataset, and each ϕ_*j*_ is the Shapley value for feature *j*. The binary variable $z_{j}^{'}$ denotes whether the feature is present. The Shapley value ϕ_*j*_ for feature *j* is formally computed as expressed in Equation 13:


$$\begin{array}{c} \phi_{j}\left(f,x\right)=\underset{S\subseteq \left\{1,\ldots ,p\right\}\backslash \left\{j\right\}}{\sum }\frac{|S|!\left(p-|S|-1\right)!}{p!}\left(f\left(S\cup \left\{j\right\}\right)-f\left(S\right)\right) \end{array}$$ (13)

The Shapley value is used to calculate the average feature contribution across all coalitions. Global feature importance determines the primary drivers of ESG scores. A beeswarm plot visualizes the distributional effects of features across model predictions. Greater transparency supports the interpretation of sustainability scores by regulators.

**Figure 6:**
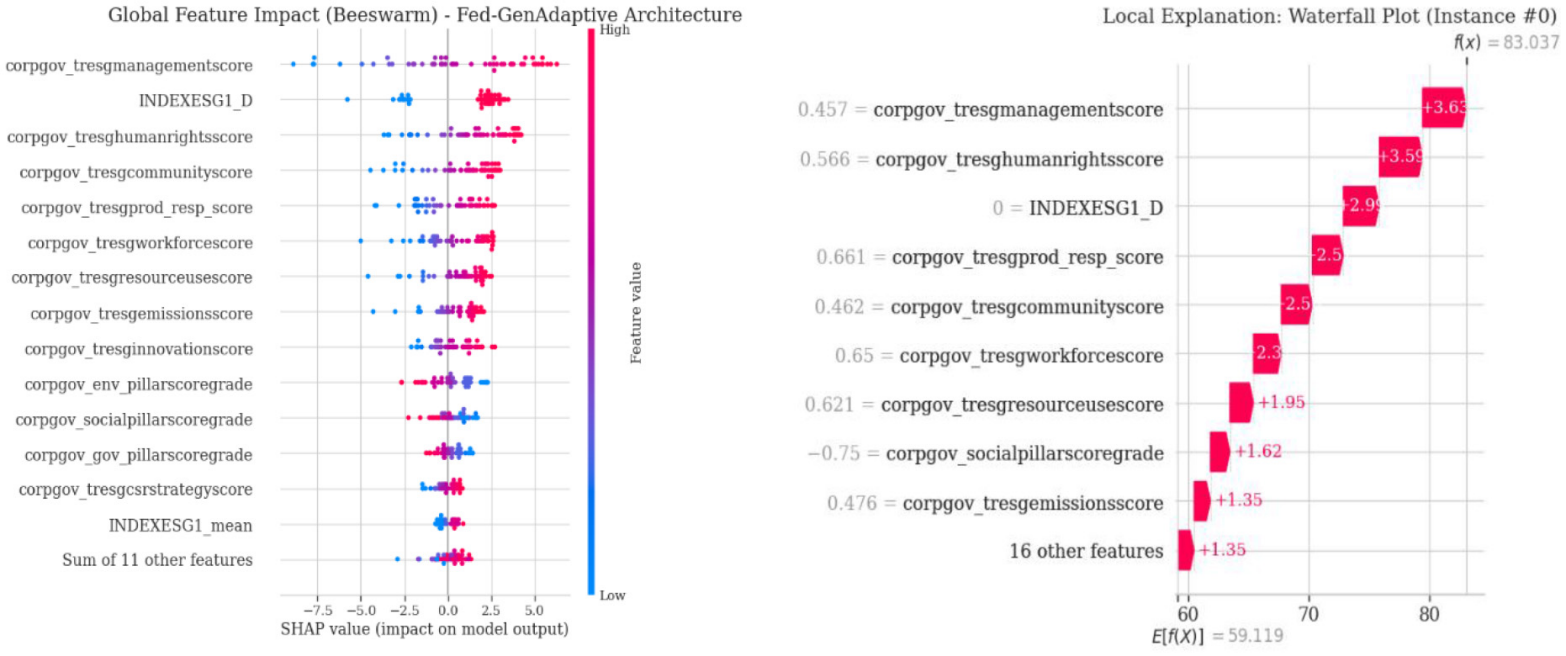
Global and local model explanations using SHAP values.

The global and local interpretations with SHAP are shown in Figure 6. The beeswarm plot highlights key drivers influencing corporate sustainability scores. Distributions represent positive and negative effects of features. The waterfall plot illustrates individual predictions for a single instance. Such tools enhance stakeholder transparency and support regulatory compliance.

### 8.2 Local interpretable model-agnostic explanations (LIME)

LIME offers local interpretability of complex model prediction results. The approach models individual predictions by locally approximating the model. Changes in prediction values are observed by perturbing the data instance. All the perturbed samples are weighted by their closeness to the original data. Such weighted local data are well fitted by a simple interpretable model. This objective function strikes a balance between model faithfulness and interpretability, which is minimized in Equation 14.


$$\begin{array}{c} \xi \left(x\right)=\arg\underset{g\in G}{\text{min}}\left[L\left(f,g,\pi_{x}\right)+\Omega \left(g\right)\right] \end{array}$$ (14)

The initial complicated model is explained using local approximations. The loss function measures the approximation error of the surrogate model. Complex surrogate models are penalized to ensure interpretable local explanations. Local linear surrogates ensure that the decision boundaries around firms are linear. When there are small corporate enhancements, the scores are predicted to rise in the framework. This proportional response incentivizes continuous investment in sustainability across industries.

**Figure 7:**
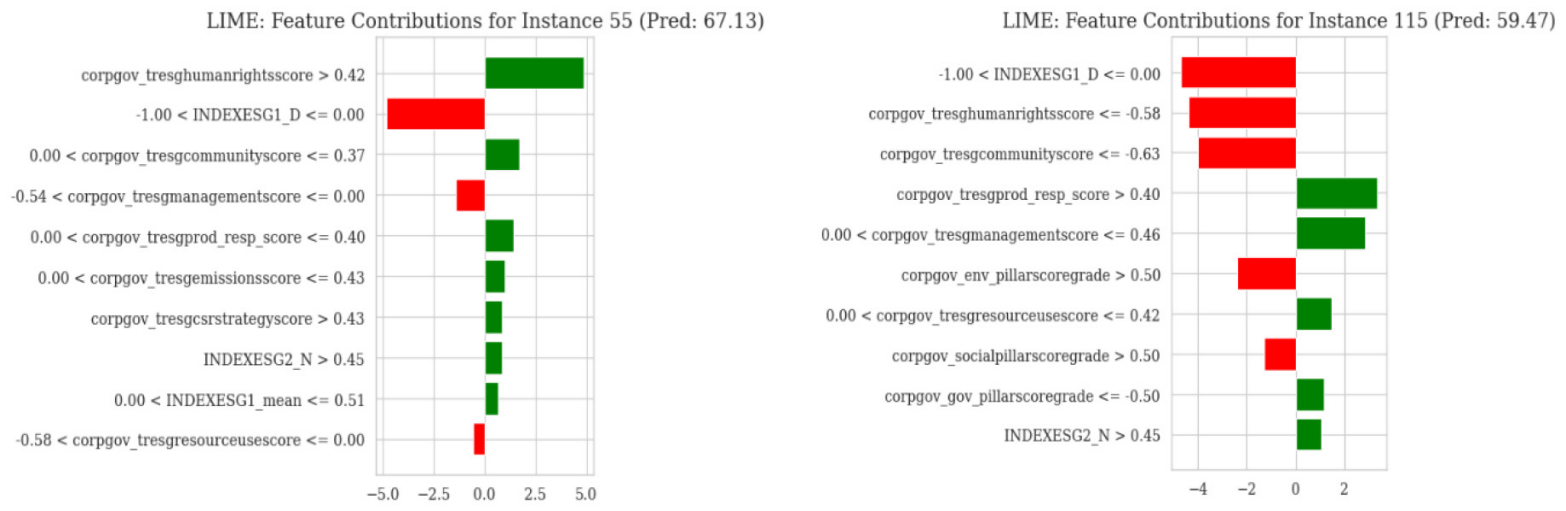
LIME-based local feature contributions for individual company predictions.

Figure 7 shows local feature contributions for each prediction case. It measures the effect of individual variables on the final audit score. Green bars indicate a positive contribution to the score, whereas red bars indicate a negative contribution. The analysis verifies the local linearity and stability of the model. These insights help auditors understand key drivers of company ratings.

### 8.3 Limitations

This study is mainly based on structured tabular data from annual reports. Relevant news and social media information are unstructured and are not utilized at present. This study examines the 10 largest industrial sectors in the international market. An increase in the number of sectors may further refine global aggregate weights. The hyperparameter λ is set to a constant of one. Dynamic tuning of this penalty parameter could be considered in future research.

The annual reporting cycle of corporations restricts the frequency of data. This delay introduces a slight lag in real-time assessment. For large-scale deployments, computational requirements for stacking multiple models may be high. The existing model presupposes that every client is adequately represented in terms of data. Missing value imputation is based on the median values of each industry sector. The aggregation weights do not explicitly capture regional differences in regulation.

The research uses a 13-year period for historical performance assessment. A longer time series could reveal additional patterns in governance trends. Three statistical tests are used to select features efficiently. Other selection methods may provide variation in predictive governance features. The application of interquartile range scaling assumes that outliers follow typical financial distributions. Preprocessing in federated data pipelines requires a common schema across nodes. In this methodology, participating clients are assumed to be honest when providing local model updates.

Furthermore, the real-world deployment of the EcoStack-Pro framework is subject to several practical constraints that warrant careful consideration. First, scalability and computational cost emerge as major challenges; although the all-tree stacking ensemble guarantees robust convergence, training three separate gradient boosting models along with a Bayesian ridge meta-learner locally requires considerable computational resources, which may strain clients with limited IT infrastructure. As the network expands to encompass hundreds of smaller sub-sectors, communication overhead and bandwidth demands are also expected to rise significantly. Second, in terms of generalizability and sector imbalance, while the Fed-GenAdaptive algorithm effectively handled an imbalance ratio of 3.5 in the dataset, highly underrepresented sectors or newly emerging industries may still face insufficient representation, potentially constraining the model's global applicability to niche markets. Finally, real-world deployment challenges such as network latency, asynchronous client updates, possible client dropouts during training rounds, and the requirement to maintain standardized data schemas across highly independent corporate entities introduce logistical barriers that must be systematically addressed before global, cross-sector ESG consortiums can fully operationalize this architecture.

### 8.4 Future work

Natural language processing of unstructured data should be included in future research. Social media sentiment analysis can provide real-time auditing feedback loops. It may be helpful to integrate the adaptive decision-making capacity with reinforcement learning. Automated hyperparameter optimization of the Fed-GenAdaptive algorithm can be explored by researchers. Global generalizability may be enhanced by increasing the dataset with emerging markets.

Rating soundness can be ensured by cross-validation with various data providers. The adoption of blockchain technology could also ensure the decentralization of data exchange. Constant training of models enables rapid adaptation to new global regulations. Examining the effect of different board structures globally should be investigated further. Environmental impact monitoring can be automated using satellite imagery integrated into data pipelines. Future research can compare this framework to more recent deep learning models. Cooperation between institutions will create a stronger international ESG consortium.

High levels of encryption will also secure sensitive financial measures during training. Practitioner user interfaces are expected to show local and global SHAP values. Audit resource allocation can be optimized by using dynamic reward functions in reinforcement learning. Long-term effectiveness of federated auditing can be evaluated using longitudinal studies. Sectors may be supported by on-site data verification using mobile auditing agents. This study opens the way to independent and open corporate governance. Internationalization of reporting will also make cross-border performance comparisons easier. Final structures will incorporate real-time information for proactive risk reduction.

## 9 Conclusion

This study investigated whether a decentralized federated architecture could reconcile the demand for universal ESG benchmarking with strict corporate data privacy constraints. The empirical analysis using the Fed-GenAdaptive framework affirms this possibility, achieving an *R*^2^ of 0.9614 and retaining 98.2% of the predictive power found in the centralized upper bound. By employing a novel soft-gating aggregation mechanism, the framework successfully neutralized the destabilizing effects of high statistical heterogeneity inherent across diverse industrial sectors. This result validates that stacked ensemble architectures can effectively surmount the “data silo” problem, delivering high-precision governance ratings without the regulatory risk of pooling sensitive proprietary information.

For the auditing profession, these findings offer a strong theoretical proof-of-concept for cross-sector ESG benchmarking under strict privacy constraints. However, realizing a global ESG consortium remains a formidable task. Future implementations must navigate immense practical challenges, including the reconciliation of highly diverse real-world data schemas, adherence to conflicting international regulatory regimes, and the design of frameworks that properly incentivize cooperation among competing financial institutions. The success of the adaptive weighting scheme demonstrates that privacy preservation does not require a significant sacrifice in analytical rigor. However, the current reliance on structured annual reporting remains a limiting factor. Future advancements must bridge the gap between retrospective reporting and real-time monitoring by integrating unstructured data, such as satellite imagery and social sentiment, into the federated pipeline, thereby shifting the focus from compliance to proactive risk reduction.

## Data Availability

The raw data supporting the conclusions of this article will be made available by the authors, without undue reservation.
